# Tirzepatide’s innovative applications in the management of type 2 diabetes and its future prospects in cardiovascular health

**DOI:** 10.3389/fphar.2024.1453825

**Published:** 2024-08-28

**Authors:** Jingqi Yang, Yuncheng Gu, Huaigang Chen, Hong Wang, Lang Hong, Bin Li, Liu Yang

**Affiliations:** ^1^ Department of Cardiology, Jiangxi Provincial People’s Hospital, The First Affiliated Hospital of Nanchang Medical College, Nanchang, Jiangxi, China; ^2^ Department of Science and Education, Jiangxi Provincial People’s Hospital, The First Affiliated Hospital of Nanchang Medical College, Nanchang, Jiangxi, China; ^3^ Jiangxi Medical College, Nanchang University, Nanchang, Jiangxi, China

**Keywords:** tirzepatide, GLP-1/GIP dual receptor agonist, glycemic management, weight control, cardiovascular health management

## Abstract

Tirzepatide, a novel GLP-1/GIP dual receptor agonist, shows significant advantages in glycemic management and weight control. By summarizing the results of the SURMOUNT and SURPASS clinical trials, we evaluate the efficacy and safety of tirzepatide in reducing blood glucose and weight. These trials indicate that tirzepatide significantly lowers HbA1c levels (with a maximum reduction of 2.24%) and promotes weight loss (up to 11.2 kg) with good tolerability. However, there are still some challenges in its clinical application, including high treatment costs and gastrointestinal discomfort. Additionally, the safety and efficacy of tirzepatide in special populations, such as patients with renal impairment, require further investigation. Future large-scale clinical trials, such as SURPASS-CVOT and SUMMIT, are expected to further verify the long-term benefits of tirzepatide in cardiovascular health management, providing stronger evidence for its comprehensive treatment of diabetes and its complications.

## 1 Introduction

Type 2 diabetes has become a major global public health challenge. According to the International Diabetes Federation (IDF) in 2021, approximately 537 million adults aged 20–79 worldwide have diabetes, which is expected to rise to 783 million by 2045 (Ogurtsova et al., 2022). Diabetes and its complications not only severely affect patients’ quality of life but also impose a heavy economic burden on individuals, families, and society. Specifically, diabetes is one of the leading causes of blindness, kidney failure, myocardial infarction, stroke, and amputation (Zheng et al., 2018).

Glucagon-like peptide-1 (GLP-1) and glucose-dependent insulinotropic polypeptide (GIP) are two important incretin hormones secreted by the intestine that play a key role in maintaining glucose homeostasis (Nauck and Meier, 2018). GLP-1 promotes insulin secretion in a glucose-dependent manner, inhibits glucagon release, delays gastric emptying, and suppresses appetite, while GIP mainly enhances glucose-stimulated insulin secretion (Holst, 2019; Nauck and Meier, 2016). GLP-1 receptor agonists, such as liraglutide and semaglutide, have become important drugs for the treatment of type 2 diabetes based on the physiological actions of GLP-1. Existing research suggests that the long-term cardiovascular benefits of GLP-1 receptor agonists (such as semaglutide) have been demonstrated through CVOT studies (Dahl et al., 2022). However, they still have limitations, such as gastrointestinal side effects (Frias et al., 2021; Buse et al., 2020). Tirzepatide, a novel GLP-1/GIP dual receptor agonist, offers a synergistic effect and demonstrates unique advantages in glucose management and weight control. This paper will systematically evaluate the efficacy, safety, and mechanisms of tirzepatide in the treatment of type 2 diabetes and explore its potential in cardiovascular disease risk management, providing a new perspective on the comprehensive management of diabetes and its complications.

## 2 Methods

### 2.1 Literature search

Retrieval should be conducted using PubMed, EMBASE, and Sino Med databases. We searched the titles of the articles using predefined keywords based on the research topic and aim. More information about the terms used in the search can be Located in Supplementary Material S1. The retrieval period spans from the inception of the data bases up to February 2024.

### 2.2 Data extraction

After independently reviewing the literature, the two researchers gathered data manually, including the type, year and results of the study. To ensure impartiality, a third researcher was selected to evaluate any differences of opinion.

## 3 Mechanisms of action and efficacy of tirzepatide

**FIGURE 1:**
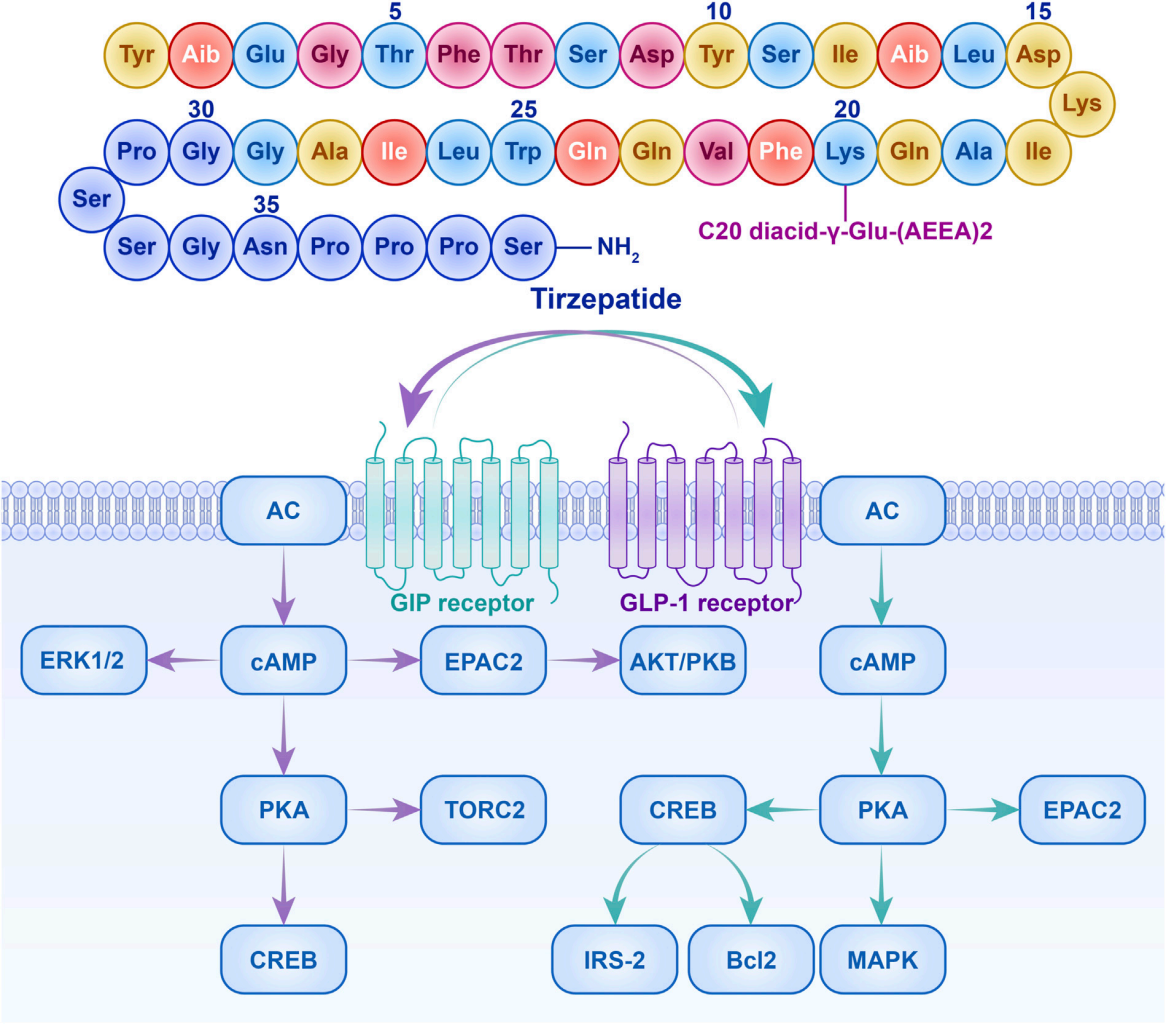
Molecular structure of tirzepatide and the dual activation mechanism of GLP-1 and GIP receptors. AC, Adenylate Cyclase; AKT/PKB, Protein Kinase B; Bcl2, B-cell lymphoma 2; cAMP, Cyclic Adenosine Monophosphate; CREB, cAMP Response Element-Binding Protein; EPAC2, Exchange Protein directly Activated by cAMP 2; ERK1/2, Extracellular signal-Regulated Kinase 1/2; IRS-2, Insulin Receptor Substrate 2; MAPK, Mitogen-Activated Protein Kinase; PKA, Protein Kinase A; TORC2, Transducer of Regulated CREB activity 2. The upper part shows the amino acid sequence of tirzepatide and its molecular structure linked to C20 diacid-γ-Glu-(AEEA)2. The lower part details the signal transduction mechanisms of tirzepatide through the activation of GIP and GLP-1 receptors (with the GIP receptor pathway shown in purple and the GLP-1 receptor pathway shown in green).

**FIGURE 2:**
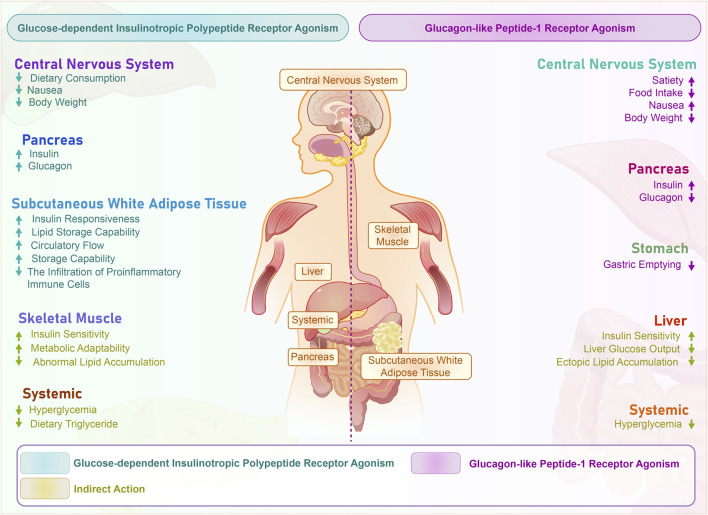
Systemic effects of tirzepatide dually activating GLP-1 and GIP receptors. The figure illustrates the impact of GIP (Glucose-dependent Insulinotropic Polypeptide) and GLP-1 (Glucagon-like Peptide-1) receptor agonism on different organs and systems, as well as their mechanism of action in metabolism. GIP receptor agonism is indicated in blue, while GLP-1 receptor agonism is indicated in purple. Direct effects are seen in the central nervous system, pancreas, and stomach, as receptors are directly stimulated. Indirect effects are mainly manifested in systemic effects, liver, and muscles, and are caused by cascade effects triggered by these direct effects. These indirect effects include improved insulin sensitivity, reduced hyperglycemia, and enhanced lipid metabolism. CNS, Central Nervous System; GIP, Glucose-dependent Insulinotropic Polypeptide; GLP-1, Glucagon-like Peptide-1.

Tirzepatide distinguishes itself from other diabetes treatments by providing a new direction for diabetes management (Willard et al., 2020). Specifically, GLP-1 receptor activation stimulates the production of cAMP, thereby promoting glucose-dependent insulin secretion by pancreatic β-cells, inhibiting glucagon secretion by α-cells, delaying gastric emptying, and suppressing the hypothalamic appetite center, resulting in glucose-lowering and weight-loss effects (Nauck et al., 2021; Svendsen and Holst, 2021; Iida et al., 2016) As shown in Figure 1. Activation of the GLP-1 receptor initiates a series of cascading reactions, which exert a hypoglycemic effect by stimulating insulin secretion from pancreatic β-cells and inhibiting glucagon secretion from α-cells, thereby reducing the stimulatory effect of glucagon on gluconeogenesis in the liver (Xia et al., 2024; Chepurny et al., 2019; Que et al., 2019; Moon et al., 2016). Meanwhile, GIP receptor activation can also enhance glucose-dependent insulin release through the AKT/PKB signaling pathway and promote β-cell proliferation while inhibiting apoptosis, playing an important role in glucose regulation and insulin secretion (Regmi et al., 2024; Mackenzie and Elliott, 2014). Finally, the dual activation of GLP-1 and GIP signaling pathways synergistically enhances insulin secretion and inhibits glucagon secretion, more effectively improving glucose homeostasis. Additionally, the dual receptor agonist may have synergistic effects in suppressing appetite and promoting satiety (Nogueiras et al., 2023). Specifically, in the central nervous system, tirzepatide can suppress appetite, promote satiety, reduce vomiting, and help with weight loss. In the pancreas, it can increase insulin secretion, inhibit glucagon secretion, and improve glucose metabolism disorders. In subcutaneous white adipose tissue, tirzepatide can increase insulin sensitivity, enhance lipid buffering capacity, increase blood flow and fat storage capacity, and reduce inflammatory cell infiltration, thus improving insulin resistance. Systemically, it can improve hyperglycemia and affect dietary triglyceride metabolism. In skeletal muscle, tirzepatide can increase insulin sensitivity, enhance metabolic flexibility, and affect ectopic lipid accumulation. In the liver, it can increase insulin sensitivity, inhibit hepatic gluconeogenesis, and affect ectopic lipid accumulation. In the gastrointestinal tract, tirzepatide can delay gastric emptying (See Figure 2).

## 4 Evaluation of the efficacy and safety of tirzepatide in the treatment of type 2 diabetes and cardiovascular risk management

### 4.1 SURPASS-1: efficacy of tirzepatide on HbA1c and weight change

The SURPASS-1 trial included 478 patients and compared the efficacy of tirzepatide at doses of 5 mg, 10 mg, and 15 mg. The primary endpoints were changes in HbA1c and body weight (Rosenstock et al., 2021). At 40 weeks, all tirzepatide doses were superior to placebo in reducing HbA1c, fasting serum glucose, and bodyweight, with more participants on tirzepatide meeting HbA1c targets. Tirzepatide induced dose-dependent bodyweight loss and had mild to moderate, transient gastrointestinal side effects, with no clinically significant or severe hypoglycaemia reported; one death occurred in the placebo group.

### 4.2 SURPASS-2 trial comparison of tirzepatide and semaglutide

The SURPASS-2 trial, comprising 1,879 patients with type 2 diabetes, compared once-weekly tirzepatide (5 mg, 10 mg, 15 mg) with semaglutide 1 mg over a 40-week period. The primary outcome assessed was the change in HbA1c levels from baseline to week 40 (Frías et al., 2021). The findings revealed that tirzepatide exhibited significantly superior efficacy to semaglutide in reducing HbA1c levels and promoting weight loss across all dosage groups, underscoring its effectiveness in managing glycemic control and weight for individuals with type 2 diabetes.

### 4.3 SURPASS-3 trial comparison of tirzepatide and insulin degludec

The SURPASS-3 trial involved 1,444 patients with type 2 diabetes who had HbA1c levels ranging from 7% to 10.5% and a BMI of 25 kg/m^2^ or higher. These patients were being treated with metformin with or without an SGLT2 inhibitor. The study compared the effectiveness of once-weekly tirzepatide at doses of 5 mg, 10 mg, and 15 mg, to once-daily titrated insulin degludec over a period of 52 weeks. The main goal was to measure the average change in HbA1c levels from the beginning to the end of the study (Ludvik et al., 2021). The findings revealed that tirzepatide outperformed insulin degludec in lowering HbA1c levels and body weight, while also resulting in fewer episodes of hypoglycemia. This indicates that tirzepatide may offer a more effective and safer treatment option for managing type 2 diabetes in this particular group of patients.

### 4.4 SURPASS-4 trial comparison of tirzepatide and insulin glargine

The SURPASS-4 trial included 1,995 patients with type 2 diabetes and compared tirzepatide (5 mg, 10 mg, 15 mg) with insulin glargine over 52 weeks, with the primary endpoint being the change in HbA1c (Heerspink et al., 2022).

### 4.5 SURPASS-5 trial on tirzepatide with insulin glargine

The SURPASS-5 trial enrolled 475 adult patients with type 2 diabetes (baseline HbA1c 7.0%–10.5%, BMI ≥23 kg/m^2^) who were on a stable dose of daily insulin glargine (>20 IU/day or >0.25 IU/kg/day). They received additional once-weekly tirzepatide (5 mg, 10 mg, 15 mg) or a placebo for 40 weeks, starting with an initial dose of 2.5 mg per week and increasing by 2.5 mg every 4 weeks. The primary endpoint was the average change in HbA1c from baseline to week 40.

### 4.6 SURPASS-6 trial: tirzepatide vs. insulin lispro

The SURPASS-6 trial included 1,428 adults with type 2 diabetes inadequately controlled by basal insulin, comparing once-weekly tirzepatide (5 mg, 10 mg, 15 mg) with thrice-daily insulin lispro. The primary endpoint was the mean change in HbA1c from baseline to week 52 for tirzepatide relative to insulin lispro (Rosenstock et al., 2023).

### 4.7 SURPASS J-mono trial in Japan

In Japan, the SURPASS J-mono trial enrolled 636 Japanese adults with type 2 diabetes (age ≥20 years) who discontinued oral monotherapy or were treatment-naive due to hyperglycemia (Yabe et al., 2023). They received tirzepatide 5 mg, 10 mg, 15 mg, and dulaglutide 0.75 mg, with the primary endpoint being the mean change in HbA1c from baseline to week 52.

### 4.8 SURPASS J-combo trial in Japan

The SURPASS J-combo trial included 443 Japanese adults with type 2 diabetes (age ≥20 years, BMI ≥23 kg/m^2^, inadequately controlled HbA1c), who received tirzepatide 5 mg, 10 mg, 15 mg in addition to oral antidiabetic drugs for ≥3 months. The primary endpoint was the incidence of treatment-related adverse events at week 52.

### 4.9 SURPASS-AP-combo trial: tirzepatide vs. insulin glargine

The SURPASS-AP-Combo trial enrolled 917 adults with type 2 diabetes (age ≥18 years, inadequately controlled by previous metformin therapy and insulin-naive), who received tirzepatide 5 mg (n = 230), 10 mg (n = 228), 15 mg (n = 229), or insulin glargine (n = 230). The primary endpoint was the mean change in HbA1c from baseline to week 40 to assess the non-inferiority of tirzepatide 10 mg and 15 mg (Gao et al., 2023).

These trials focused on evaluating the efficacy of tirzepatide in glycemic control and weight management, providing an effective treatment option for patients with type 2 diabetes and opening new possibilities for future cardiovascular health management. Table 1 summarizes the design and main results of these key trials.

**TABLE 1:** Summary of key clinical trial designs and primary endpoints for tirzepatide.

Trial name	Population	Inclusion criteria	Dosage regimen	Primary endpoint
SURMOUNT-1	2,539	Adults with an average weight of 104.8 kg and an average BMI of 38.0	Once-weekly tirzepatide (5 mg, 10 mg, 15 mg) or placebo for 72 weeks	The percentage change in weight from baseline to week 72 and the proportion of participants achieving a weight reduction of 5% or more at week 72
SURPASS -1	478	Patients with T2D	Tirzepatide 5, 10, 15 mg, QW	HbA1c and weight change
SURPASS -2	1,879	Patients with T2D	Once-weekly tirzepatide (5, 10, 15 mg) or semaglutide 1 mg	Change in HbA1c from baseline to 40 weeks
SURPASS -3	1,444	HbA1c 7%–10.5%, BMI ≥25 kg/m2, treated with metformin ± SGLT2 inhibitor	Once-weekly tirzepatide (5 mg, 10 mg, 15 mg) or once-daily titrated insulin degludec for 52 weeks	52-week, mean change from baseline HbA1c
SURPASS -4	1,995	Patients with T2D	Tirzepatide (5 mg, 10 mg, 15 mg) or insulin glargine for 52 weeks	Change in HbA1c
SURPASS-5	475	Adult type 2 diabetes patients, baseline HbA1c 7.0%–10.5% (53–91 mmol/mol), BMI ≥23 kg/m^2^, on stable once-daily insulin glargine (>20 IU/day or >0.25 IU/kg/day), with or without metformin (≥1,500 mg/day	Tirzepatide (5 mg, 10 mg, 15 mg) or placebo for 40 weeks, with tirzepatide initiated at 2.5 mg once weekly and escalated by 2.5 mg every 4 weeks	mean change in baseline glycated hemoglobin A1c (HbA1c) at week 40c
SURPASS-6	1,428	Patients with type 2 diabetes mellitus in adulthood who have insufficient control of basal insulin	Tirzepatide (5 mg, 10 mg, 15 mg) once weekly or insulin lispro thrice daily	At week 52, the mean HbA1c change comparing Tirzepatide with insulin Lispro
SURPASS J-mono	636	Adult patients in Japan with type 2 diabetes mellitus, aged 20 and above, who have discontinued oral monotherapy for hyperglycemia or are treatment-naive patients	Tirzepatide 5, 10, 15 mg and dulaglutide 0.75 mg	52-week, mean change from baseline HbA1c
SURPASS J-combo	443	Adult patients in Japan with T2D (age ≥20 years, BMI ≥23 kg/m^2^, inadequately controlled HbA1c)	Tirzepatide 5 mg, 10 mg, 15 mg in addition to oral antidiabetic drugs for ≥3 months	Incidence of treatment-related adverse events at week 52
SURPASS-AP-Combo	917	Adults (≥18 years old) with type 2 diabetes (T2D) who are uncontrolled with metformin (with or without sulfonylureas) and have not been treated with insulin before	Tirzepatide 5 mg (n = 230), 10 mg (n = 228), 15 mg (n = 229), or insulin glargine (n = 230)	mean change in HbA1c from baseline to week 40 to assess the non-inferiority of 10 mg and 15 mg tirzepatide
SURMOUNT-CN	210	Obese or overweight adult patients in China	Tirzepatide (10 mg, 15 mg) or placebo once weekly for 52 weeks	Superiority of Tirzepatide (10 mg/15 mg) over placebo in weight change at 52 weeks; proportion achieving ≥5% weight loss

T2D, type 2 diabetes; CV, cardiovascular; QD, once daily; QW, once weekly; FPG, fasting plasma glucose; PPG, postprandial glucose; SGLT2, Sodium-Glucose Co-Transporter-2; IU, International Unit.

### 4.10 SURMOUNT-1: tirzepatide for weight loss in obese adults

In the SURMOUNT-1 trial, 2,539 adults with an average weight of 104.8 kg and an average BMI of 38.0 were treated with weekly doses of tirzepatide (5 mg, 10 mg, 15 mg) or placebo for 72 weeks. The primary endpoints were the percentage change in body weight relative to baseline and the proportion of patients achieving a weight loss of 5% or more (Garvey et al., 2023).

### 4.11 SURMOUNT-CN: tirzepatide for weight loss in Chinese obese and overweight adults

The SURMOUNT-CN trial recruited 210 obese or overweight adult patients in China, who received weekly doses of tirzepatide (10 mg, 15 mg) or placebo for 52 weeks. The primary endpoints were the superiority of tirzepatide over placebo in terms of weight change at 52 weeks and the proportion of patients achieving a weight loss of ≥5%.

## 5 Potential applications and prospects of tirzepatide in cardiovascular health

### 5.1 Effects of tirzepatide on cardiovascular risk factors: possible theoretical basis

Tirzepatide, as an innovative drug for the treatment of type 2 diabetes, not only improves glycemic control and promotes weight loss but may also have a positive impact on cardiovascular health. In clinical trials, tirzepatide significantly reduced HbA1c levels (by up to 2.24%) and promoted substantial weight loss (by up to 11.2 kg) (Frias et al., 2021; Frias et al., 2018). These effects may translate into cardiovascular benefits.

**FIGURE 3:**
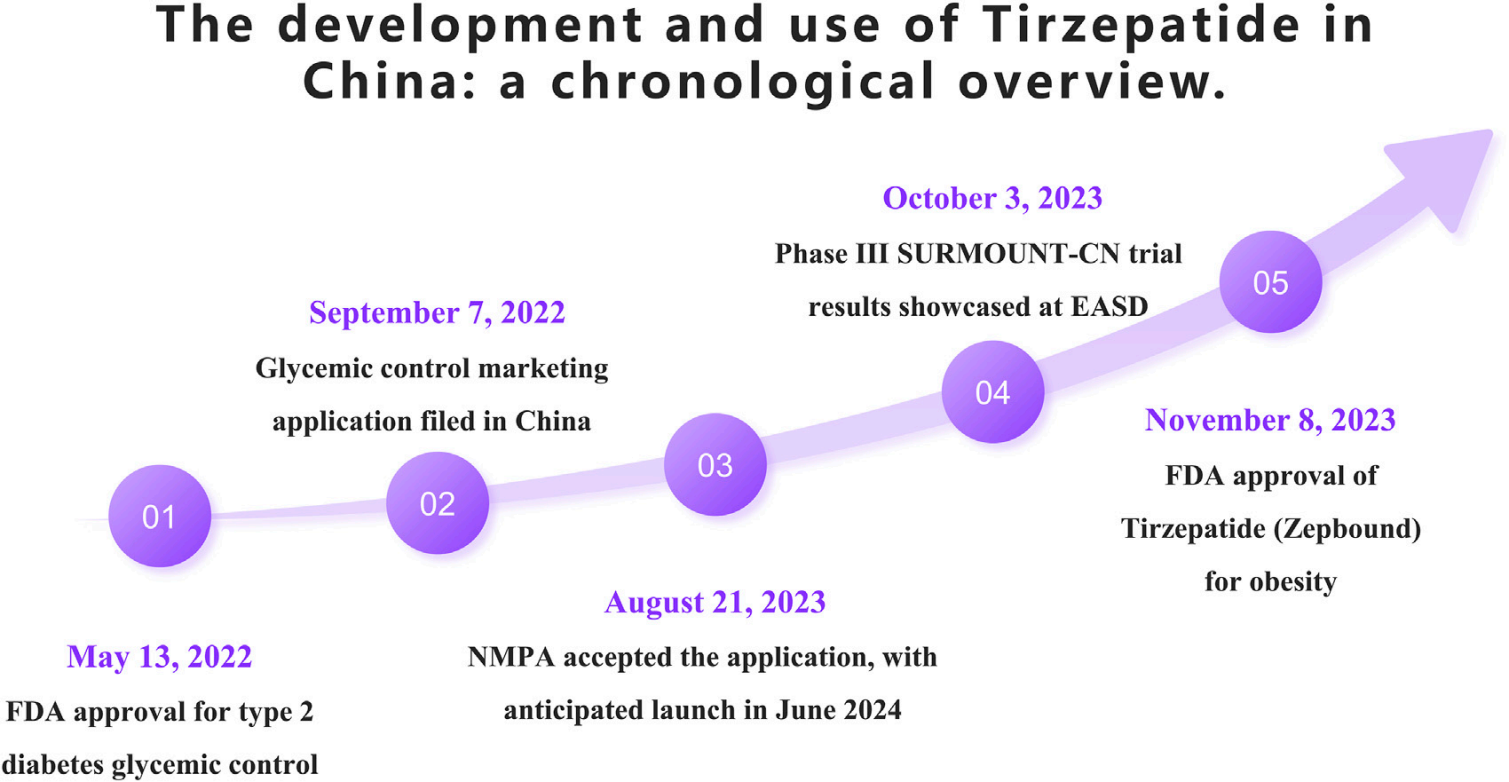
The development and use of Tirzepatide in China: a chronological overview.

Molecular Mechanisms: Tirzepatide may positively impact cardiovascular health through multiple mechanisms (see Figure 3). Firstly, it improves glycemic control by reducing HbA1c levels, thus mitigating the vascular damage caused by hyperglycemia. High blood glucose levels or glucose fluctuations can lead to vascular endothelial dysfunction and atherosclerosis through mechanisms such as increased oxidative stress, the formation of advanced glycation end products (AGEs), and the activation of protein kinase C (PKC) (Brownlee, 2001; Dubsky et al., 2023; Torimoto et al., 2013). Additionally, tirzepatide may help lower blood pressure and reduce cardiovascular burden through direct effects such as improving vascular endothelial function and indirect effects via weight management. Some studies suggest that GLP-1 receptor agonists may have direct cardiovascular protective effects beyond their benefits on glucose lowering and weight loss. For example, in animal models, liraglutide and semaglutide have shown anti-atherosclerotic effects, including reducing vascular inflammation, improving endothelial function, and stabilizing plaques (Rakipovski et al., 2018; Tashiro et al., 2014). However, although the research on tirzepatide is still in the preliminary stage, there is some data to support its potential benefits in cardiovascular protection (see Section 5.2 Prospective Clinical Trial Progress and Results of Tirzepatide in the Treatment of Type 2 Diabetes with Cardiovascular Disease for details). Moreover, in a hyperglycemic ApoE gene knockout mouse model, liraglutide was found to inhibit atherosclerosis through an AMPK-independent mechanism, suggesting that GLP-1 receptor agonists may exert cardiovascular protective effects through multiple mechanisms (Koshibu et al., 2019). These effects may be partially mediated by anti-inflammatory and antioxidant mechanisms driven by the GLP-1 receptor.

Preliminary evidence also suggests that tirzepatide may confer cardiovascular benefits by improving lipid metabolism (e.g., lowering LDL cholesterol) and reducing inflammation (e.g., lowering C-reactive protein). For instance, in an 8-week randomized controlled trial, tirzepatide treatment significantly reduced LDL cholesterol and C-reactive protein levels in patients with type 2 diabetes (Del et al., 2021). However, these findings need further validation in larger and longer-term clinical trials.

Given that tirzepatide activates both GLP-1 and GIP signaling pathways, its cardiovascular benefits may arise not only from the effects of GLP-1 but also from the potential effects of GIP. For example, in ApoE-deficient mice, GIP analogs significantly reduced atherosclerosis and improved vascular function (Tashiro et al., 2014). However, research on the cardiovascular effects of GIP is still relatively limited, and the similarities and synergistic effects between GIP and GLP-1 require further exploration (Cho et al., 2023; Fisman and Tenenbaum, 2021). Particularly, according to the 2019 ESC guidelines and the 2019 ADA/EASD consensus report, GIP and GLP-1 signaling pathways can synergistically improve cardiovascular risk factors, but their long-term cardiovascular benefits still need to be confirmed through further research (Lim et al., 2023).

### 5.2 Prospective clinical trial progress and results of tirzepatide in the treatment of type 2 diabetes with cardiovascular disease

Currently, two significant cardiovascular outcome trials for tirzepatide are underway (or have recently concluded), including SURPASS-CVOT and SUMMIT. SURPASS-CVOT is a large-scale, randomized, double-blind, multinational phase III clinical trial that enrolled 13,299 Type 2 Diabetes Mellitus patients with increased cardiovascular risk. It aims to compare the safety and efficacy of tirzepatide versus dulaglutide in preventing major adverse cardiovascular events (MACE). The trial is expected to be completed in October 2024 (Nicholls et al., 2024). The SUMMIT trial focuses on adult patients with heart failure with preserved ejection fraction (HFpEF) and obesity. To date, 731 participants have been enrolled to compare the long-term effects of tirzepatide versus placebo in improving major adverse cardiovascular events (MACE), weight, and metabolic indicators, and to assess its efficacy in enhancing exercise tolerance in heart failure patients. Secondary objectives include evaluating the effects of tirzepatide on weight, cardiac structure, and function. This trial is still ongoing. See Table 2 for detailed information.

**TABLE 2:** Overview of ongoing tirzepatide clinical trials for cardiovascular and metabolic conditions.

Trial name	Start date	Due date	Study subjects	Trial design	Comparator drug	Primary outcome measures
SURPASS-CVOT	May 2020	OCT 2024	Type 2 Diabetes Mellitus patients with increased cardiovascular risk	Interventional, randomized, double-blind, parallel assignment	Tirzepatide vs. Dulaglutide	The CV effect of tirzepatide versus dulaglutide on time To first occurrence of MACE
SUMMIT	April 2021	June 2024	Adults with Heart Failure with Preserved Ejection Fraction (HFpEF) and Obesity	Randomized,Double-Blind, Placebo-Controlled, Phase 3 Study	Tirzepatide vs. Placebo	Change in KCCQ Clinical Summary Score and Incidence of Cardiovascular Death or Heart Failure Events Over Time

CVOT, cardiovascular outcomes trial; KCCQ, kansas city cardiomyopathy questionnaire; HFpEF, heart failure with preserved ejection fraction.

The results of these two trials will provide more comprehensive and higher-quality evidence for the application of tirzepatide in cardiovascular disease management, which is of great significance for guiding clinical practice. Based on the existing evidence, these ongoing trials are expected to further expand our understanding of the cardiovascular effects of tirzepatide, providing stronger support for its use in the integrated management of patients with type 2 diabetes and cardiovascular disease.

## 6 The applications of tirzepatide in China mainland nowadays

While Tirzepatide has been developed and approved in Western markets, its introduction to the Chinese market is still pending. The drug is anticipated to be offered in six strengths-2.5, 5, 7.5, 10, 12.5, and 15 mg. According to the official pricing information provided by Eli Lilly (https://pricinginfo.lilly.com/mounjaro), the list price of Mounjaro, the brand name for Tirzepatide, per ‘fill’ in the USA is $1,069.08, where ‘fill’ represents a monthly supply consisting of four weekly injections. The high cost and lack of availability in China, where Tirzepatide has not yet been launched or included in medical insurance schemes, could pose significant barriers to access. These factors emphasize the need for strategies to manage costs and engage in insurance negotiations to improve accessibility once the drug becomes available. Assessing Tirzepatide’s cost-effectiveness in China will be crucial as real-world application data becomes available following its market entry.

### 6.1 Tirzepatide’s benefits for Chinese patients

In the near future, Tirzepatide is expected to be more widely available across various cities and hospitals in China, which could significantly enhance the quality of life for many Chinese residents. It is anticipated that millions of Chinese patients suffering from type 2 diabetes or obesity, who are not effectively managed with current treatments, will benefit from this drug.

### 6.2 Tirzepatide’s entry into China

**FIGURE 4:**
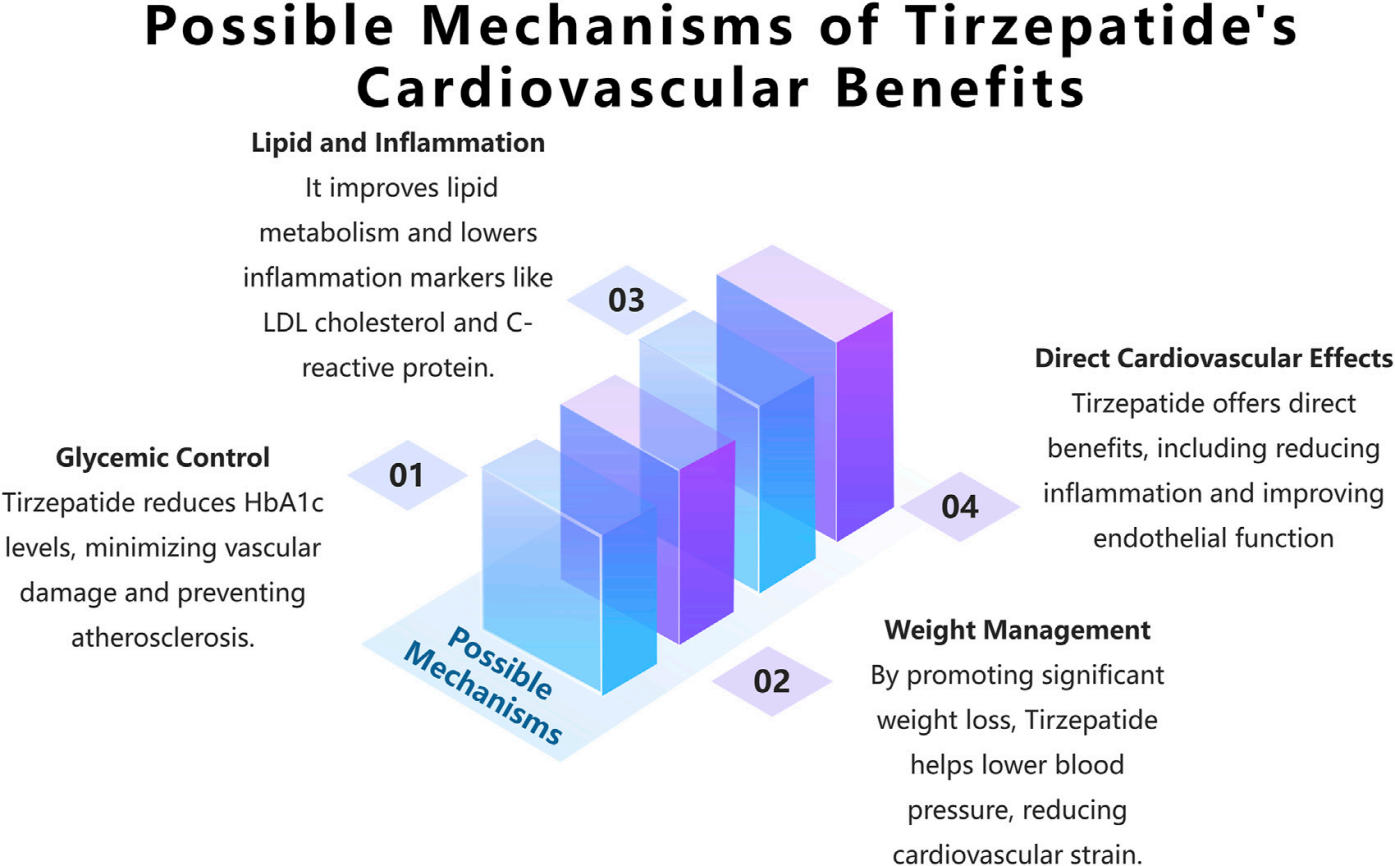
Possible mechanisms of Tirzepatide’s cardiovascular benefits.

On 13 May 2022, the FDA approved Tirzepatide as an adjunct therapy for glycemic control in adults with type 2 diabetes. Later that year, on September 7, Tirzepatide’s application for glycemic indication was submitted for approval in China. The Chinese National Medical Products Administration (NMPA) officially accepted this application on 21 August 2023, with an anticipated market launch in mid-June 2024. On 3 October 2023, during the European Association for the Study of Diabetes (EASD) annual meeting, Eli Lilly presented results from the Phase III SURMOUNT-CN clinical trial conducted in China, which enrolled 210 patients. The study demonstrated that Tirzepatide was superior to placebo in reducing weight. On 8 November 2023, Tirzepatide, branded as Zepbound in the US, received FDA approval as the first dual-target agonist for the treatment of obesity, further highlighting its potential in medical treatment options. The following timeline (Figure 4) summarizes the main events and dates of the introduction and utilization of Tirzepatide in China.

## 7 Discussion

In the modern management of type 2 diabetes, tirzepatide, as a novel drug with a unique therapeutic mechanism, has attracted widespread attention. This review discusses its comprehensive impact, from diabetes control to potential cardiovascular benefits, and explores its role as part of an integrated treatment regimen. Additionally, it analyzes major clinical trials in the current literature, including its efficacy in different patient populations, treatment challenges, and future research directions. By summarizing data from key trials such as the SURPASS and SURMOUNT series, this review aims to provide clinicians with the latest evidence-based information on the comprehensive application of tirzepatide to guide higher-quality clinical decision-making.

Undoubtedly, most trial data come from studies with sufficiently large sample sizes and diverse populations. However, there are also smaller trials, such as SURMOUNT-CN with 210 cases, where conclusions might be influenced by the smaller sample size. Furthermore, while the majority of clinical trial designs have reasonable endpoints, there are also non-inferiority analyses like SURPASS-AP-Combo.Studies have shown that tirzepatide excels in improving glycemic control and promoting weight loss. Results from the SURPASS and SURMOUNT series trials suggest significant efficacy in lowering blood glucose (with reductions of up to 2.24%) and in weight loss (up to 11.2 kg). This significant weight loss not only helps improve metabolic parameters in diabetes but may also bring additional benefits to cardiovascular health.

Comparisons with semaglutide indicate that tirzepatide has an advantage in reducing HbA1c and body weight. This could be attributed to its dual GLP-1 and GIP receptor agonist mechanism, which enhances metabolic regulation. Moreover, the once-weekly administration of tirzepatide not only improves patient compliance but also simplifies the treatment regimen, making it more advantageous for long-term treatment (as seen in SURPASS-2).

Additionally, comparisons with insulin degludec and insulin glargine show that tirzepatide performs better in reducing HbA1c and body weight, while also reducing hypoglycemic events. This advantage may stem from tirzepatide’s multiple mechanisms of action, including promoting insulin secretion and suppressing appetite, leading to excellent performance in both glycemic control and weight management (as observed in the SURPASS-3 and SURPASS-4 trials).

Regarding injection frequency, tirzepatide’s once-weekly injection method shows significant advantages in weight loss and glycemic control, improving patient compliance more effectively than daily injections, such as liraglutide. The once-weekly dosing frequency not only offers convenience for patients but also more effectively manages blood glucose levels and weight. Furthermore, the combination use of tirzepatide with basal insulin has shown significant effects in optimizing blood glucose management.

In the SURPASS-5 and SURPASS-6 trials, the combination of tirzepatide with insulin glargine or insulin lispro significantly improved HbA1c levels while reducing both hyperglycemic and hypoglycemic events. The combination strategy leverages the multiple mechanisms of tirzepatide and the stable glucose-lowering effect of basal insulin, providing a comprehensive management plan for diabetes patients, thereby maximizing treatment efficacy (as demonstrated in SURPASS-5 and SURPASS-6).

Trials have shown that tirzepatide is generally well-tolerated, with common adverse reactions being gastrointestinal symptoms. Most of these were mild to moderate and occurred mainly during dose escalation. The rates of discontinuation due to adverse events in the 5 mg, 10 mg, and 15 mg tirzepatide groups and the placebo group were 4.3%, 7.1%, 6.2%, and 2.6%, respectively (Jastreboff et al., 2022). Additionally, other weight-loss diabetes medications seem to have more adverse reactions (Papamargaritis et al., 2024; Wilding et al., 2021). Possible explanations for this include differences in administration methods and frequencies. Liraglutide requires daily subcutaneous injections, while semaglutide and tirzepatide are administered once weekly. This difference in dosing frequency may lead to variations in the incidence of adverse reactions. Although most side effects are mild to moderate and improve over time, they can still impact patient compliance. Targeted management strategies include starting with a low dose and gradually increasing it, taking the medication after meals to reduce gastrointestinal discomfort, and optimizing diet (e.g., avoiding high-fat foods) (Wharton et al., 2022). During the initial treatment phase, providing patient education and psychological support can also help improve drug tolerance and satisfaction. However, long-term safety data, especially regarding cardiovascular safety, still need further accumulation. The main adverse reactions reported so far include gastrointestinal symptoms such as nausea, vomiting, and diarrhea (H et al., 2024). These symptoms are usually more obvious at the beginning of treatment and alleviated over time. It is recommended to gradually increase the dose (Frías et al., 2019) or take the drug after meals to reduce these adverse reactions. Furthermore, there are other negative responses that may occur, including skin rashes where the injection was given. Nevertheless, the majority of these reactions are mild to moderate and can be managed by patients (Mizumoto, 2023).

Furthermore, in the comprehensive treatment of type 2 diabetes with tirzepatide, we still face multiple challenges. One challenge is accurately identifying which patients are most suitable for tirzepatide. This requires a comprehensive assessment of the patient’s clinical characteristics, such as the duration of diabetes, presence of comorbidities, body mass index, and response to previous treatments. For example, the benefits for overweight/obese individuals were highlighted in the SURMOUNT-1 trial, which involved overweight/obese individuals with an average weight of 104.8 kg and a BMI of 38.0, indicating that tirzepatide could be a new option for weight loss in this specific population.

Secondly, the high cost of tirzepatide treatment (approximately tens of thousands of dollars per year) may limit its widespread use in economically constrained regions (such as mainland China). As we all know, the high cost of reality mainly comes from the research and development, marketing, and patent protection of original drugs. In the future, several potential solutions may be implemented, including the establishment of a robust governmental medical insurance department, the formulation of a national negotiation strategy for the original pharmaceutical company, and the potential creation of a patient assistance program. Notably, in 2024, the China National Medical Insurance Negotiation Plan is anticipated to incorporate this particular drug. In the near future, improved insurance coverage and patient assistance programs will help increase the accessibility of tirzepatide.

Additionally, patients with renal impairment are another special group that requires attention. Tirzepatide is primarily excreted by the kidneys, so reduced renal function could theoretically affect its pharmacokinetics and efficacy (Velenosi and Urquhart, 2014). However, a pharmacokinetic study of tirzepatide in patients with moderate renal impairment (eGFR 30–59 mL/min/1.73 m^2^) indicated that reduced renal function does not significantly alter the exposure or glycemic effects of tirzepatide (Urva et al., 2021). This could be due to the structural characteristics of tirzepatide, which confer a longer half-life, reducing the impact of decreased drug clearance due to renal impairment, maintaining relatively constant blood concentrations, and ensuring sustained glycemic control. Despite the increased risk associated with older patients due to diminished physical capabilities and the presence of multiple comorbidities, research has consistently demonstrated favorable efficacy and tolerability in this demographic. A particular study indicates that tirzepatide markedly enhances glycemic control in older adults (mean age at study enrollment: 60.6 years) with type 2 diabetes who exhibit inadequate glycemic regulation following insulin glargine therapy. Additionally, the study reports a significant weight reduction effect associated with tirzepatide (Dahl et al., 2022).

Finally, there may be differences in drug metabolism, absorption, and tolerance among different ethnic groups, which may affect the efficacy and safety of tirzepatide. In addition, lifestyles and eating habits of different regions and ethnic groups may also affect the efficacy of tirzepatide. For example, factors such as diet and exercise habits may change the patient’s response to the drug. Therefore, future studies should further explore these differences to ensure the applicability and safety of tirzepatide in different populations, and consider the influence of lifestyle factors on efficacy to optimize the therapeutic effect in different populations.

Although preliminary studies have shown that tirzepatide has good efficacy and safety in different ethnic groups, further studies are still needed to fully understand ethnic differences and their impact on drug efficacy in order to develop more personalized treatment strategies.

## 8 Conclusion

Tirzepatide, an innovative treatment for type 2 diabetes, has demonstrated significant effects on glycemic control and weight management through the dual activation of GLP-1 and GIP receptors. Its weight loss effects were notably significant in the SURMOUNT and SURPASS trials. However, future research needs to conduct long-term follow-up and large sample studies to more comprehensively evaluate the cardiovascular effects of tirzepatide, validating its potential as a safe and effective treatment option for diabetes and heart disease. Additionally, research on special populations, such as elderly patients and those with renal impairment, should not be overlooked.

## 9 Prospects

Future research should focus on the long-term safety and efficacy of tirzepatide, especially its role in cardiovascular disease management. The latest SURPASS-CVOT and SUMMIT trials are expected to provide in-depth insights, comparing the effects of tirzepatide with other treatments like dulaglutide. These research findings will help provide safer and more effective treatment options for patients with type 2 diabetes and may redefine prevention strategies for cardiovascular disease.
