# Supplementary material for: Tirzepatide’s innovative applications in the management of type 2 diabetes and its future prospects in cardiovascular health
Source: Front Pharmacol. 2024 Aug 28;15:1453825. doi: 10.3389/fphar.2024.1453825 (PMC11387164; doi:10.3389/fphar.2024.1453825)
Supplement: Supplementary file 1 [file Table1.DOCX]

In this study, we used the following keywords and search strategies to search for literature,

*Tirzepatide[Title/Abstract]*

*GLP-1/GIP dual receptor agonist [Title/Abstract]*

*(Tirzepatide[Title/Abstract]) AND (Glycemic management[Title/Abstract])*

*(Tirzepatide[Title/Abstract]) AND (Weight control[Title/Abstract])*

*(Tirzepatide[Title/Abstract]) AND (Cardiovascular health management[Title/Abstract])*

*(GLP-1/GIP dual receptor agonist [Title/Abstract]) AND (Glycemic management [Title/Abstract])*

*(GLP-1/GIP dual receptor agonist [Title/Abstract]) AND (Weight control [Title/Abstract])*

*(GLP-1/GIP dual receptor agonist [Title/Abstract]) AND (Cardiovascular health management [Title/Abstract])*

*(Tirzepatide[Title/Abstract]) AND (GLP-1/GIP dual receptor agonist[Title/Abstract])*

*(Tirzepatide[Title/Abstract]) AND (Clinical Trials[Title/Abstract])*

*(Tirzepatide[Title/Abstract]) AND (Adverse Events[Title/Abstract])*

*(GLP-1/GIP dual receptor agonist [Title/Abstract]) AND (Clinical Trials [Title/Abstract])*

*(GLP-1/GIP dual receptor agonist [Title/Abstract]) AND (Adverse Events [Title/Abstract])*

*Tirzepatide Filters: from 2010 - 2024*

*(Tirzepatide) OR (GLP-1/GIP dual receptor agonist) Filters: from 2010 - 2024*
